# Femtosecond electron microscopy of relativistic electron bunches

**DOI:** 10.1038/s41377-023-01142-1

**Published:** 2023-05-11

**Authors:** Yang Wan, Sheroy Tata, Omri Seemann, Eitan Y. Levine, Slava Smartsev, Eyal Kroupp, Victor Malka

**Affiliations:** Department of Physics of Complex Systems, Weizmann Institute of Science, Rehovot, 7610001 Israel

**Keywords:** Plasma-based accelerators, Laser-produced plasmas, Imaging and sensing

## Abstract

The development of plasma-based accelerators has enabled the generation of very high brightness electron bunches of femtosecond duration, micrometer size and ultralow emittance, crucial for emerging applications including ultrafast detection in material science, laboratory-scale free-electron lasers and compact colliders for high-energy physics. The precise characterization of the initial bunch parameters is critical to the ability to manipulate the beam properties for downstream applications. Proper diagnostic of such ultra-short and high charge density laser-plasma accelerated bunches, however, remains very challenging. Here we address this challenge with a novel technique we name as *femtosecond ultrarelativistic electron microscopy*, which utilizes an electron bunch from another laser-plasma accelerator as a probe. In contrast to conventional microscopy of using very low-energy electrons, the femtosecond duration and high electron energy of such a probe beam enable it to capture the ultra-intense space-charge fields of the investigated bunch and to reconstruct the charge distribution with very high spatiotemporal resolution, all in a single shot. In the experiment presented here we have used this technique to study the shape of a laser-plasma accelerated electron beam, its asymmetry due to the drive laser polarization, and its beam evolution as it exits the plasma. We anticipate that this method will significantly advance the understanding of complex beam-plasma dynamics and will also provide a powerful new tool for real-time optimization of plasma accelerators.

## Abstract (web summary)

The first direct, non-invasive characterization of the lateral structure of a relativistic, micro-sized electron bunch generated from a laser-plasma accelerator.

## Graphical abstract

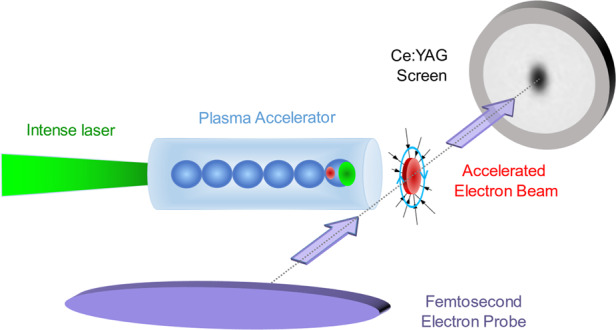

## Introduction

Over the past four decades, laser-plasma accelerators (LPAs) have achieved astonishing progress worldwide and are considered as one of the leading candidates for future accelerators^1–7^. In these compact devices, an intense short laser pulse is focused into a gaseous plasma and excites a strong wake developing during its propagation^8^. Since the plasma medium is ionized it is free of breakdown and can easily sustain an extraordinarily large accelerating gradient greater than 100 GV/m, more than 1000 times stronger than that achievable in conventional accelerators. Such plasma wakes have been demonstrated to accelerate electrons over a distance of only few centimeters up to several GeV^9–11^ with femtosecond bunch duration^12^, micrometer-scale source size^13^ and ultralow emittance^14^. Therefore, LPAs have shown great potential in many rapidly emerging applications including ultra-short drivers for compact undulator synchrotron sources^15–17^, free-electron-lasers (FELs)^7^, and multi-stage accelerators for high-energy physics^6,18^. Albeit promising, the electron sources from such devices have raised technical difficulties for coupling them from the plasma into these downstream applications. In order to efficiently transport the beam without spoiling its quality, accurate information of the beam parameters just near the plasma exit is highly required.

Previously, the temporal duration of the LPA electron bunch was characterized as few femtoseconds using techniques such as coherent transition radiation^12,19^, ultrafast Faraday rotation^20^ and optical streaking^21^. On the contrary, their transverse properties were mainly measured at positions far from the plasma source using quadrupole scans^14,22,23^ and pepper-pot masks^24–26^. Other methods relied on the spectra analysis of x-ray Betatron or Compton radiations^13,27,28^ only give an indirect estimate of the beam diameter either averaged over the whole acceleration region^13,27^ or after entirely outside the plasma^28^. This, however, is insufficient for advanced applications that rely on the manipulation of the beam at a close proximity to the plasma source, where it is much more difficult to directly access the instantaneous information of the beam. No technique, to date, has yielded accurate information about the beam’s lateral profile and its evolution inside the plasma. Understanding these dynamics is crucial for many on-going applications, for instance, for uses that require coupling the electron beam between different accelerator or radiator components^6,7,15–17,29,30^.

## Results

**Fig. 1:**
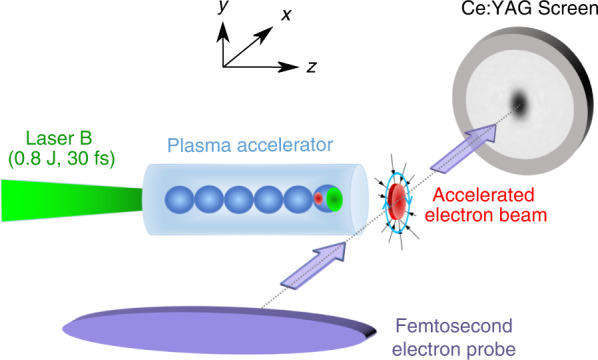
Schematic of the experimental setup. A femtosecond electron bunch (the small red circle), was generated from a laser-plasma accelerator created by focusing Laser B onto a 5-mm diameter gas jet. After reaching the rear side of the gas jet, the bunch expanded laterally into a larger size (the large red disk), and was probed by utilizing another electron beam generated from the same type of laser-plasma accelerator created by Laser A (not shown in the figure). The probe beam was deflected by the electron bunch’s space- charge field (E field indicated as black straight arrows and B field indicated as blue curved arrows). The probe density modulation after a 13 mm drift was recorded by a 30 µm-thick Ce:YAG screen, followed by a high-resolution imaging system. The resulted hollow structure on the imaging screen resembles the investigated electron beam’s lateral profile as explained in the Methods and Materials

In this work, we present the first direct, instantaneous measurements of LPA electron beams’ lateral charge density profiles at different positions along the plasma exit (see Fig. 1). The concept involves a second LPA electron bunch as a probe which has been demonstrated to have few- femtosecond temporal and micrometer spatial resolutions^31,32^. In this concept, the probe electrons get deflected by the space-charge fields of the investigated beam, when crossing it in the transverse direction. After a short drift, these deflections evolve into a spatial density modulation that contains transient information of the beam’s field distributions.

### Femtosecond electron microscopy of the LPA bunch

To validate the proposed method, we performed the experiment at the HIGGINS dual 100 TW laser platform of the Weizmann Institute of Science^33^. The LPA-generated electron bunch that was investigated was obtained by focusing a 0.8 J, 30 fs laser beam onto a 5-mm diameter supersonic gas jet using a mixture of helium and nitrogen with a plasma density of 4 × 10^18^ cm^−3^ in the plateau. Averaged over 40 consecutive shots in the energy spectrometer, the obtained electron bunch had a quasi-monoenergetic spectrum with the peak energy at 152 ± 18 MeV and around 45% FWHM relative spread. The overall charge above 50 MeV was 92 ± 33 pC. (More details can be found in Methods and Materials, and also Fig. S1 of the Supplementary). Independently, a second intense laser pulse of 1.5 J, 30 fs was focused into an 8 × 10^18^ cm^−3^ density plasma generated from a supersonic gas jet composed of the same gas mixture. This produced a stable electron probe beam, which contained a peak energy of 380 ± 40 MeV and 20–25% FWHM spread. After exiting the jet and drifting for 10 cm in vacuum, the probe beam typically spatially expanded to several hundreds of micrometers. It then intercepted the investigated electron bunch and its trailing plasma wakes, obtaining additional spatially dependent transverse momenta. After another drift of 13 mm, the probe beam with developed density modulation impinged on a thin Ce:YAG scintillating screen and was imaged. In the experiments, both lasers were linearly polarized in the horizontal plane. For convenience of the geometry, we define the *z*-axis as the investigated electron bunch propagation direction, *x* as the probe beam propagation direction and *y* as the vertical direction. More details about the laser system and experimental setup can be found in the “Materialsand methods”.

**Fig. 2:**
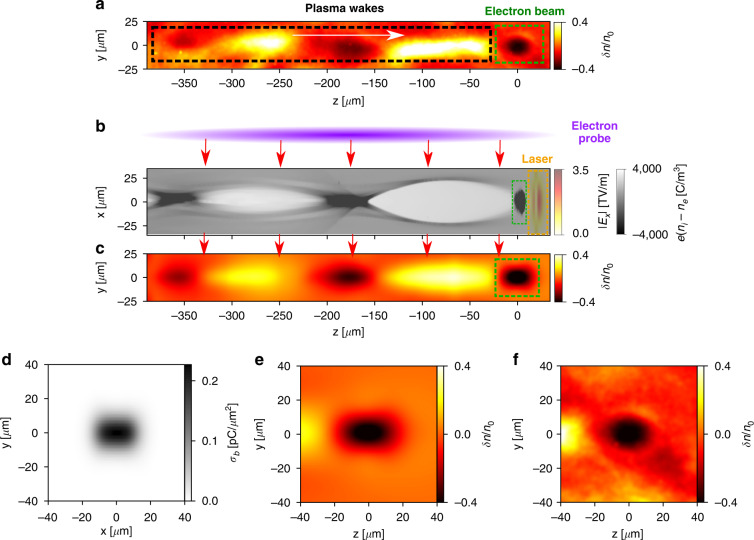
Snapshot of the electron beam and associated plasma wakes at 5 mm after the jet center and the comparison with PIC simulations. **a** Experimental image of the probe relative density with background subtracted. **b** Simulated plasma charge density *en*_*p*_ and laser electric field *E*_*x*_ distributions at the moment when the probe crosses them. **c** Simulated image of the probe relative density at an imaging position of 10 mm. **d** The transverse profile of the electron bunch accelerated in the simulation shown in (**b**). **e** Simulated probe image of the electron bunch extracted from (**c**). **f** Experimental probe image of the electron bunch extracted from (**a**)

Figure 2a shows one experimental probe image taken at the position where the investigated electron bunch is about 5 mm after the jet center. In the image, $\delta n/n_0 = \left( {n - n_0} \right)/n_0$ represents the relative density modulation of the probe, with *n* the actual probe density measured from experiment, and *n*_0_ the density background obtained by smoothing the raw data with a low-pass Gaussian filter^32,34^. One can see a hollow elliptical shape followed by a periodic bright-dark pattern propagating from left to right. The trailing structure is observed to have a transverse size close to the elliptical structure but with an extended longitudinal size of around 210 μm for the first period. On the other side, the elliptical shape, marked by the green dashed box, correlates with the occurrence of an electron bunch in the energy spectrometer. Therefore, we interpret the probe image pattern to be a relativistic electron bunch driving its own plasma wake along the plasma density downramp. To verify this hypothesis, relativistic three-dimensional particle-in-cell (3D PIC) simulations^35^ have been performed to simulate the entire process, where the input laser parameters and gas type were the same as in the experiment and the gas density profile was extracted from computational fluid dynamic (CFD) simulations using the same nozzle design for accurately mapping the density information over 2 cm range. More details about the PIC simulation setup can be found in the “Materials and methods”.

From the PIC simulation, it is shown that after self-focusing^36,37^ and self-compression^38,39^ in the first few-mm propagation, the laser pulse excites a nonlinear plasma wake and electrons ionized from the inner shell of nitrogen are trapped and accelerated in the first bucket^40,41^. At the rear downramp of the gas jet, since the laser intensity gets much weaker due to depletion and diffraction, the wakefield launched by the accelerated electron bunch starts to be dominant. This phenomenon is shown in Fig. 2b, where the simulated plasma charge density *en*_*p*_ and laser electric field *E*_*x*_ distributions at the same position as Fig. 2a are presented. One can see a quasi-linear plasma wave launched by an electron bunch (marked by the green dashed box) and ahead of it, the laser pulse (marked by the orange dashed box) is already below relativistic intensity. By directly comparing the on-axis longitudinal electric field it is found that the strength of the laser driven wakefield is almost one order lower than that of the bunch driven wakefield, whereas the residual laser intensity is still sufficient for ionizing the gas ahead of the bunch. At this moment and position, an electron probe, propagating along the *x* direction, interacts with the z-propagating electron bunch. The simulated probe image of Fig. 2c shows similar structures to the ones observed in experiment (Fig. 2a), revealing the transient field information imprinted in the probe density variations. In the following, we focus on the elliptical structure that mainly results from the space-charge force generated by the LPA electron bunch.

It is known that the typical size of an electron bunch inside the plasma accelerator (usually over the density plateau region) is micrometer scale^13,27^. However, near the plasma exit, the millimeter-range density downramp can easily expand the bunch’s size to several micrometers because of the gradually weakening of the transverse focusing forces from the wakefield^25^. This was investigated numerically in many previous studies^14,42,43^ and is also confirmed in our simulation, where the lateral size of the electron bunch (see Fig. 2d) at the probed position is already far above one micrometer. On the other side, for the conditions described above, the electron bunch from LPA normally has a duration of a few femtoseconds^12,20,21^ (2 fs obtained from our simulation), much smaller than its transverse size. Based on the above analysis, the shape of electron bunch at the downramp region in our condition can be considered to have a shape similar as an ultra-thin disk and we integrate the associated fields along the probe particles’ paths. It turns out that the elliptical structure of the probe density modulation directly correlates with the lateral profile of the investigated bunch (see Eq. 1 and the relevant content in the Methods and Materials) and this can be verified by the comparison between Fig. 2d, e, which shows that the investigated electron bunch’s lateral profile (*x*–*y* plane) is directly imprinted in the probe image (*y*–*z* plane), while its duration contributes only marginally. To further validate this point, we have changed the probe interception direction from the *x* to the *y* axis in another simulation, and the corresponding elliptical shape in the probe image also rotated by 90° (see Fig. S2 of the Supplementary).

To quantitatively estimate the absolute charge distribution of the investigated bunch, we need to take into account all relevant fields that contribute to the probe modulation of the elliptical structure. After detailed analysis, it is found that besides the bunch self-fields that play the major role, the plasma fields also impact the probe deflections in two manners. First, the probe electrons traversing the bunch also move through the trailing linear wakefield and gain additional momenta (see Section I of the Supplementary). Second, the space-charge fields of the bunch are partially screened by the induced plasma lateral currents^44,45^, which generate opposing electromagnetic fields over the small length of the drive bunch (see Section II of the Supplementary). Both of these effects counteract the repulsive force from the bunch fields.

Based on the analytical model presented in the “Methods and materials”, we now estimate the investigated electron bunch’s parameters based on the magnified probe image in Fig. 2f. For simplicity, assuming that the probe beam has the same energy spectrum over the density modulation region and fitting the observed elliptical hollow with a bi-Gaussian distribution, the electron bunch is calculated to has a peak lateral surface charge density of *σ*_*b*0_ = −0.16 ± 0.017 pC µm^−2^ with RMS radii of Δ_y_ = 7.5 µm (vertical) and Δ_x_ = 12.5 µm (horizontal). The overall charge of absolute value contained in the bunch is then 94 ± 10 pC. This value is slightly higher than the one 85 ± 17 pC calculated from the calibrated Lanex scintillator for the same shot, perhaps due to the fact that the low-energy electrons below 50 MeV were not collected by the energy spectrometer. We also note that these low-energy electrons usually have large divergences and lateral sizes at the probed position, and therefore might not be fully included in our above analysis if their distributions extend outside the assumed bi-Gaussian bunch profile.

To further validate our analytical model and compare directly with the experimental data, we have performed another numerical simulation implementing a probe beam traversing an electron bunch with the above parameters that propagates in a plasma of the density 2.5 × 10^16^ cm^−3^ (corresponding to a plasma wavelength 210 µm of Fig. 2a). Based on the probe density modulation after 13 mm drift, the reconstructed bunch contains a charge of 98 pC, very close to the input value 94 pC, while its transverse sizes are slightly larger than the actual ones (by around 10%) because of the trajectory expansion of the probe particles. More details about the simulation can be found in section III of the Supplementary.

### Bunch profile dynamics at the plasma exit

**Fig. 3:**
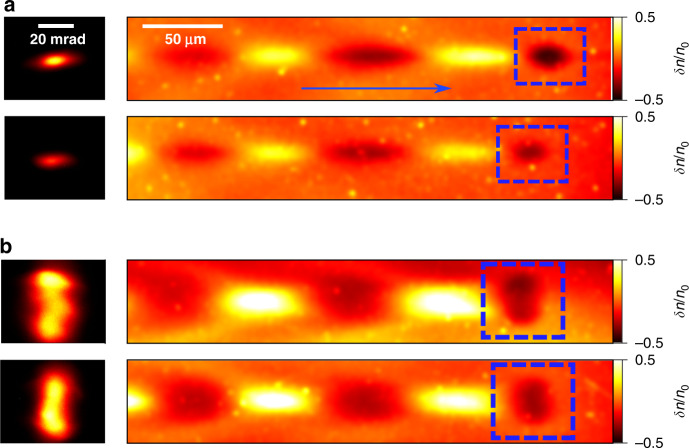
Experimental observation of the beam asymmetry due to laser polarization. The experimental results of the probe images (right) and corresponding electron bunch angular profiles (left) for linear (**a**) and elliptical (**b**) laser polarization. For each case, two consecutive shots are presented, where the electron bunch angular profiles (left) were measured by a Lanex scintillator and its spatial shape before exiting the plasma were captured by the probe images (right)

Let us now illustrate the application of this concept to uncover important features of the laser-plasma accelerated bunch as it leaves the plasma. The first distinctive feature is the beam asymmetry produced by the laser polarization that affects the shape of electron beam produced via ionization injection. For this study we used a quarter wave-plate inserted in the laser path before the final focusing optics, allowing the laser polarization to be tuned. In addition to the probe imaging system, a Lanex scintillator was put 300 mm after the gas jet, shielded by an 800-µm-thick copper foil to block the residual laser light and very low-energy electrons. This allowed for the simultaneous recording of the electron beam angular profiles. Figure 3 presents the measured results for linear (a) and elliptical (b) polarization respectively. From two consecutive shots for each case, it follows that the electron beam far-field (left sub-figures) and near-field profiles (marked by the blue dashed box in the right sub-figures) have very similar shapes and that they both vary with the laser polarization. In the linear case they are strongly elongated along the polarization (horizontal) axis, while for the elliptical polarization they increase moderately along the horizontal axis and dramatically along the other axis. We note that the resulted elliptical polarization, which was supposed to be circular (defined by the wave-plate), was due to the nonlinear optical effects caused by the high-intensity laser pulse when passing through the plate.

The underlying physics of Fig. 3 can be interpreted by considering the electron dynamics during the ionization injection process^46^. It is known that the majority of the electrons trapped in this process come from the inner shell of the nitrogen which requires a very intense field for ionization. Thanks to the conservation of canonical momentum, after interacting with the laser field, electrons usually gain large residual momenta orientated preferentially along the polarization axis (laser electric field direction)^47^. Since these electrons are injected at different moments along the laser propagation direction, they oscillate in different phases and frequencies, leading to the filling up of the entire phase space^48^. As a result, the whole accelerated bunch presents a significant increase in both its divergence and source size along the laser polarization axis. The experimentally observed correlations show very good agreement with this interpretation.

**Fig. 4:**
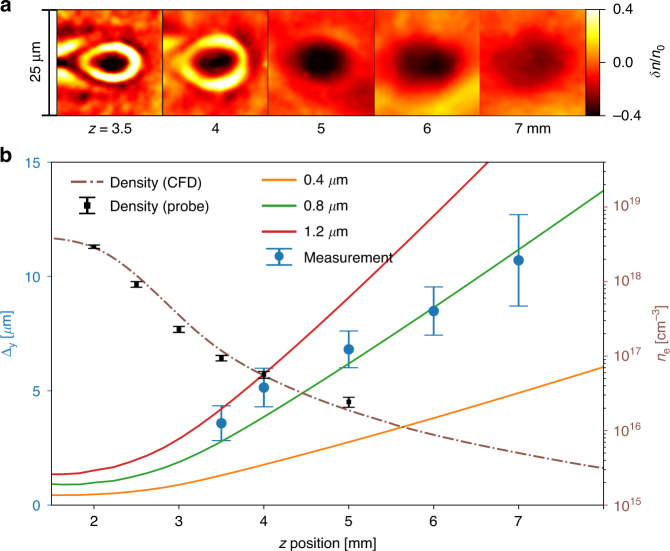
Experimental observation of the evolution of the electron beam envelope at the plasma to vacuum transition. **a** Experimental snapshots of the electron beam at five different *z* positions. **b** The measured vertical beam RMS sizes ∆_*y*_ (blue solid circles, averaged over 4–8 shots) and corresponding simulated values for different initial beam sizes (solid lines) are plotted as a function of propagation distance away from the jet center along the plasma density downramp. In simulation, the applied density profile was extracted from CFD simulation (brown dashed line) and was confirmed with the observed wakefield periods from the probe images averaged over 3 shots (black solid squares). The bunch transverse shape was taken to be Gaussian with RMS size ∆_*y*_ as indicated by the legend. Other bunch parameters used as input for the simulation were experimentally measured mean values

Let us now consider the evolution of an LPA electron beam as it exits the accelerator. Proper understanding of and control over such beam dynamics, which depend on the gas density profile and the electron beam parameters, is crucial for the efficient matching of the electron bunch into another plasma accelerator stage or into downstream radiator components^42,43^. By probing the electron beam along the plasma-vacuum transition, we have, for the first time, observed the corresponding evolution of the beam envelope. Figure 4a presents five probe image snapshots of the electron beam at different longitudinal positions, where *z* = 0 is defined as the gas jet center and the propagation is in the positive *z* direction. One can see that the transverse size of the beam keeps increasing as it propagates out of the plasma. In Fig. 4b, we plot the estimated beam vertical RMS radius Δ_y_ as a function of the *z* positions, which quantitatively shows a nearly linear relation (solid blue circles), corresponding to a slope of $d{\Delta}_y/dz \simeq$ 1.96 mrad. This value is comparable to the bunch final divergence 2.2 ± 0.3 measured in the far-field for the same shots. The slight mismatch possibly come from the fact that the bunch is still confined by the plasma fields instead of free drift in vacuum^14^ and that the stronger probe density modulation will lead to the more enlargement of the reconstructed bunch size due to trajectory expansion of the probe particles.

The obtained description was compared to 3D PIC simulations using the density profile extracted from CFD modeling (dashed brown line). Such a density profile in the downramp region was also cross-checked with the wakefield periods at several longitudinal positions based on the obtained probe images (black solid square)^20,32,49^. In these simulations we considered three cases with the electron bunches externally injected into the plasma wakes at *z* = 1 mm, with the energy spectra and charges similar as experiments but with different initial transverse sizes. The relative distance between the bunch and the laser was carefully chosen to ensure the bunch energy was not modified significantly. The best agreement in the beam envelope evolution is found for an initial size of 0.8 µm RMS (green solid line), while the cases with 0.4 and 1.2 µm sizes deviate greatly from the experimental data. In the performed simulations, it is also shown that the bunch’s divergence was reduced considerably before *z* = 4.5 mm, indicating that in this region the laser wakefield is still sufficient to focus the bunch as a laser-plasma lens^50^. This argument was further confirmed by an additional simulation with an externally injected electron bunch containing much lower charge of 1 fC, which avoided the effect of the beam driven wakefield and showed very similar evolution of the bunch size. Besides, we have also numerically checked the influence of the density profile on the beam evolution using a sharper density downramp which presented much faster beam expansion as expected.

In conclusion, for the first time, we have successfully applied femtosecond ultrarelativistic electron microscopy to directly image and characterize an LPA electron bunch using another LPA beam within femtosecond accuracy. This non-destructive diagnostic is able to capture the transient beam envelope inside a plasma accelerator, yielding information about the absolute charge density. We expect this new technique to provide guidance for real-time monitoring and fine-tuning of the beam parameters for many future applications.

## Materials and methods

### Generation and characterization of the two LPA electron beams

The experiment was carried out using the HIGGINS dual 100 TW laser system at the Weizmann Institute of Science^33^. The HIGGINS system is comprised of two separate laser pulses (A and B) split from a 7 J laser. These two pulses are independently compressed to around 30 fs duration, delivered to the target chamber and focused down to 28.1 µm and 29 µm spots (FWHM) respectively.

The electron probe beam was generated by focusing Laser A (~1.5 J on target) onto a super- sonic flow from a 3 mm diameter nozzle, where the gas was a mixture of around 99% helium and 1% nitrogen. Peak plasma density along the laser path was 8 × 10^18^ cm^−3^ as measured by an offline Mach-Zehnder interferometer and assuming complete ionization. The accelerated electrons had a quasi-monoenergetic spectrum with a peak energy of 380 ± 40 MeV and 20–25% FWHM energy spread. Their angular RMS divergences were 4.2 ± 0.8 mrad in the horizontal direction and 1.8 ± 0.4 mrad in the vertical direction and the total charge contained in the monoenergetic peak was 140 ± 40 pC. More information about the probe spectra and angular profiles can be found in ref. ^31^.

The investigated electron bunch was generated by focusing Laser B (~0.8 J on target) onto a supersonic flow from a 5 mm diameter nozzle of the same gas mixture. Mach-Zehnder interferometric measurements and CFD simulations (ANSYS FLUENT) showed that the gas density profile contained an ~3.5 mm plateau surrounded by ~1.2 mm gradients on each side, providing a peak electron density of around 4 × 10^18^ cm^−3^ after full ionization. The investigated electron bunch was measured to have a peak energy of 152 ± 18 MeV with around 45% FWHM relative spread. The total charge and vertical divergence for energies above 50 MeV were 92 ± 33 pC and 2.3 ± 0.45 mrad, respectively. Images of experimental data for the electron energy spectra can be found in Fig. S1 of the Supplementary.

The data statistics of probe and investigated electron beams’ charge, energy spectra and divergence were measured using 40 consecutive shots respectively before performing the actual pump-probe experiment and the error bars come from shot-to-shot fluctuation, Lanex calibration uncertainty^51^ and measurement errors such as divergence-induced position uncertainty in the energy spectrometer.

### Synchronization of the two electron beams

The probe electron beam, after exiting the first gas jet, drifted in vacuum for around 10 cm and intercepted the investigated electron beam at the downstream ramps of the second gas jet. At that point, the probe beam was laterally spread over several hundred micrometers. In order to spatially and temporally overlap these two beams, lasers A and B were first overlapped in the second gas jet by using pulse A for shadowgraphy of the ionization front produced by pulse B. After that, a large-view, low-resolution imaging system was used to directly overlap the electron probe with the wakefield created by either Laser B or the investigated electron beam.

### High-resolution imaging system

The probe beam profile, with variations created by the investigated bunch, was recorded onto a 30 µm-thick Ce:YAG scintillating screen placed 13 mm away from the second jet with a 100-µm-thick stainless steel foil in front of it. The image on the screen was then transmitted by a combination of a long working-distance plan-apochromatic microscope objective and an achromatic lens with *f* = 200 mm to a 16-bit SCMOS camera. ZEMAX simulations were used to validate the performance of the imaging system. A series of point sources uniformly located along the optical axis was used to simulate the light emission from the crystal. The generated point-spread function (PSF) presented a similar pattern as the ideal Airy disk but with slightly stronger side lobes, and the RMS width was estimated at about 0.6 µm after fitting with a Gaussian function. In addition, the scattering effect of electrons from the protection foil and the YAG crystal was also evaluated using Monte Carlo Geant4 simulation, which exhibited an RMS size of around 0.4 µm.

### Particle-in-cell simulations

Numerical modeling was done using the pseudo-spectral code FBPIC^35^ with a quasi-cylindrical geometry simulated with *N*_*m*_ = 5 azimuthal modes. In the simulation presented in Fig. 2 of the manuscript and Fig. S2 of the Supplementary, the cylindrical simulation box had 450 µm length and 160 *µ*m radius, and was resolved with a mesh of ∆*z* = 40 nm and ∆*r* = 320 nm. The laser driver (pulse B) was initialized with Gaussian temporal and spatial profiles of 30 fs (FWHM) duration, 29 *µ*m (FWHM) spot size at focus, and a normalized amplitude of *a*_0_ = 1.1. The gas profile was extracted from the CFD simulation with the same mixture. All electrons from the helium atoms and five electrons from the L shell of the nitrogen atoms were set pre-ionized, providing a peak electron density of *n*_*e*_ = 4 × 10^18^ cm^−3^. In this simulation, the modeling window first co-propagated with the drive laser, and we recorded the simulation states (i.e. checkpoints) at multiple positions along the propagation. These checkpoints allowed us to re-start the simulation at these positions self-consistently with a stationary modeling window and with added probe particles from one transverse boundary. The probe bunch had a *τ*_probe_ = 3 fs duration, with a peak energy of 380 MeV and 20% FWHM energy spread. The probe normalized emittance was set as 2 mm mrad (estimated from PIC simulation) and its divergence was the same as in the experiment, and the number of macro-particles in the bunch was chosen to be 4 × 10^7^.

In the simulation presented in Figs. S3–5 of the Supplementary, the cylindrical simulation box had 250 µm length and 150 µm radius with *N*_m_ = 5 azimuthal modes, and was resolved with a mesh of ∆*z* = 100 nm and ∆*r* = 200 nm. The investigated electron bunch was initialized with Gaussian temporal and spatial profiles of 3 fs RMS duration, 12.5 µm RMS radius along the horizontal direction and 7.5 µm RMS radius along the vertical direction. It had a peak energy of 150 MeV and 45% FWHM energy spread and the overall charge was 94 pC. The plasma density was 2.5 × 10^16^ cm^−3^. The probe electron beam was launched to propagate along the horizontal direction and the beam characteristics were set to be the same as in the simulation mentioned above.

In the simulations presented in Fig. 4 of the manuscript, the cylindrical simulation box was 60 µm in length and 150 µm in radius, and was resolved with a mesh of ∆*z* = 40 nm and ∆*r* = 160 nm. The laser driver (pulse B) was initialized with the same parameters as the simulation shown in Fig. 2. The gas density profile was extracted from the CFD simulation and consisted of pure helium to avoid ionization injection. In each simulation, an electron bunch with a different source size (at focus) but the same energy spectrum and charge as in the simulation of Figs. S3–5 was externally injected into the first wake period at a position of 1 mm after the jet center. The beam divergence was set to match the focusing strength of the wakefield, $\theta _ \bot = {{{\mathrm{{\Delta}}}}}_ \bot {{{k}}}_{{{\mathrm{p}}}}/\sqrt {2\left\langle \gamma \right\rangle }$ with Δ_⊥_ the source bunch size inside plasma, *k*_*p*_ the plasma wave number in the plateau and 〈*γ*〉 the averaged Lorentz factor of the bunch^52^.

### Estimation of the beam self-fields based on the probe density modulation

An LPA electron beam with the above parameters generally has a pulse duration of a few femtoseconds, significantly smaller than its lateral sizes at the density downramp, as validated by the presented simulation and experimental results in Fig. 2. Therefore, we assume it has the shape of a very thin disk with a Gaussian longitudinal profile of an RMS size, δ*z*, much smaller than its transverse RMS sizes, Δ_*x*,*y*_. Moving at a velocity close to light speed in vacuum, *c*, this beam generates localized electromagnetic fields (i.e., *E*_*x,y*_, *B*_*x,y*_) around itself (see Fig. 1 of the manuscript). After an ultra- relativistic probe bunch with an initial momentum p_0_ and zero duration crosses these fields along the x-axis and drifts a further distance of L, the distribution of the produced density modulation on the (y, z) plane can be obtained as:

1 $$I_{\rm{beam}}\left( {y,z} \right) = \frac{{\delta n}}{{n_0}} \simeq \kappa \epsilon _0\nabla \cdot \mathop {\int}\limits_{ - \infty }^{ + \infty } {\left( {E_y\left( {z - x,x,y} \right) + cB_y\left( {z - x,x,y} \right)} \right)dx \simeq {\upkappa}\sigma _b\left( {y,z} \right)}$$

Where $\kappa = eL/Mcp_0\epsilon _0$ is the coefficient with *e* the electron charge, *c* the light speed in vacuum, *ϵ*_0_ the vacuum permittivity and *M* the geometric magnification (*M* = 1.13 in our experiment). σ_*b*_ represents the lateral surface charge density of the investigated bunch if substituting *z* with *x*. The contribution of *B*_*y*_ is included in the electric field through *cB*_*y*_ = *E*_*x*_ based on the superposition property of the beam self-fields.

Besides the dominant bunch fields, the plasma fields provide the second-order contributions through two approaches. The first is the linear wakefield driven by the bunch, and the second is the plasma lateral currents induced screening fields. Consequently, the total probe density modulation can be simply expressed as,

2 $$I_{\rm{total}}\left( {y,z} \right) = I_{\rm{beam}} + \widetilde {I_{\rm{wake}}} + I_{\rm{screen}}$$

where $\widetilde {I_{\rm{wake}}}$ and *I*_screen_ are modulation caused by the linear wakefield and plasma screening fields respectively. Based on the linear wakefield theory and the assumption of $k_p\delta _z \ll 1$ and tri-Gaussian bunch distribution for simplicity, the formulas of $\widetilde {I_{\rm{wake}}}$ and *I*_screen_ can be derived analytically (see section I and II of the Supplementary). Our analysis shows that with regard to the probe electrons, the plasma fields contradict the repelling force from the bunch, and thus the bunch charge would be underestimated without considering these effects.

### The influence of non-ideal probe beams

The theoretical model in the previous section is based on an ideal probe beam. In reality, several effects from the probe such as energy spread, pulse duration and emittance can blur the created density modulation and need to be considered. All these has been previously discussed in Ref. ^53^ for probing plasma wakes. Since their effects on probing electron bunch are quite similar, here we discuss briefly about each factor only from theoretical aspect.

First, assuming the probe with a temporal profile of *τ* (*t*), the measured result *I*_meas_ is simply a one-dimensional convolution of the actual result *I*_real_ and *τ*(*t*), i.e., ${\int}_{ - \infty }^\infty {I_{\rm{real}}\left( {y,z - ct} \right)\tau \left( t \right)dt}$ which inevitably enlarges the horizontal scales of the features and reduces the image contrast while the vertical scales remain unchanged. As discussed earlier, the electron beam generated from an LPA typically has a duration of a few femtoseconds (2 fs in our PIC simulation), which is considerably smaller than the observed scale size of the hollow structure (e.g. *>*3 *µ*m) and therefore only contribute marginally to the field estimation.

Second, the probe beam normally has an energy spread and here we define its normalized energy distribution as *f*(*E*). The generated density modulation thus becomes, $I_{\rm{meas}} = {\int}_{ - \infty }^{ + \infty } {I_{\rm{ideal}}\left( E \right)f\left( E \right)dE}$ with *I*_ideal_ the density modulation from a monoenergetic beam with the energy *E*. For simplicity, we assume the probe have a flat-top energy spectrum with a peak energy of *E*_0_ and a FWHM spread of Δ*E*, the above integral is thus simplified into, $I_{\rm{meas}} \simeq \left( {1/\eta } \right)\ln \left( {2 + \eta } \right)/\left( {2 - \eta } \right)I_{\rm{real}}\left( {E_0} \right)$, where *η* = Δ*E*/*E*_0_ represents the relative energy spread. Based on the formula, it is apparent that even with very large energy spread such as 50%, the derivation of the probe density modulation is only 2.2%. For other energy distributions such as sin^2^ or Gaussian, the integral can be numerically calculated and the derivation of the density modulation for a 50% FWHM energy spread is still less than 4%. Therefore, the spectra width 20–25% of our probe will not have significant impact on the data analysis. In contrast, the fluctuation of the probe peak energy is more severe to the data analysis (see Eq. 1), which need to be considered for the uncertainty of the field estimation.

Third, the trace emittance of the probe beam can be assumed to have the form of $\epsilon _t = \sigma _0\theta _0$ for simplicity, with σ_0_ the initial probe beam size and θ_0_ the probe beam divergence. After drifting in vacuum for a distance of *D*, the probe encounters the investigated electron bunch and the wakefield. At this position, the probe beam already laterally spreads into a much larger size as *Dθ*_0_ for $D\theta _0 \gg \sigma _0$, while its slice angular spread for each transverse position reduces to $\epsilon _t/D\theta _0 = \sigma _0/D$ as a rough estimate. This slice angular spread could create imaging blurring, and the resulted resolution is estimated as $L\sigma _0/D$ with *L* the drifting distance after traversing the investigated bunch. The normalized emittance of LPA electron beams is typically 1–2 mm mrad, which gives the beam initial size as few micrometers. Considering the configure of our experiment (*L* = 13 mm and *D* = 100 mm), the blurring scale is only O(0.1) µm, much smaller than the observed scale size of the hollow structure (e.g. *>*3 µm) and therefore the emittance effect is also negligible.

Since the probe energy spread and the emittance in our experiment do not affect the final result considerably, we neglect them for simplicity. We assume both the probe and probed electron bunches with a Gaussian temporal profile of 3 fs RMS duration and a standard error of 1 fs and take into account the image resolution including optical PSF and foil scattering, the investigated bunch lateral charge distribution is calculated after the data deconvolution process. The error bar of each estimate arises from probe energy fluctuation, duration uncertainty and shot-to-shot variation if averaged over several shots.

## Supplementary information

Suppmentary information

## Data Availability

All data in the main text or the extended data materials are available upon reasonable request from the corresponding authors.
