# Supplementary material for: Femtosecond electron microscopy of relativistic electron bunches
Source: Light Sci Appl. 2023 May 11;12:116. doi: 10.1038/s41377-023-01142-1 (PMC10172298; doi:10.1038/s41377-023-01142-1)
Supplement: Supplementary file 1 — Suppmentary information [file 41377_2023_1142_MOESM1_ESM.pdf]

Supplementary Information for
Femtosecond electron microscopy of relativistic electron bunches

Yang Wan,* Sheroy Tata, Omri Seemann, Eitan Y. Levine,
Slava Smartsev, Eyal Kroupp, and Victor Malka
Department of Physics of Complex Systems,
Weizmann Institute of Science, Rehovot 7610001, Israel

I. THE EFFECT OF THE LINEAR PLASMA WAKEFIELD ON PROBE DENSITY MODULATION

As shown in Fig. 2a of the manuscript, the investigated electron bunch is observed driving its own plasma wake along the density downramp. When the probe electrons traverse the electron bunch, they also pass through the trailing wakefield. Even though the strength of the wakefield is more than one order lower than the space-charge fields of the drive bunch, the probe path length across the wakefield is longer than that across the electron bunch, which could effectively reduce the probe deflection. To estimate the wakefield contribution, it is required to estimate its field strength.

Through 3D PIC simulations, it is shown that the plasma wave launched by an electron bunch with the same parameters as experiment is under quasi-linear regime (see Fig. S3 and section III). From the wakefield image captured by the probe (see Fig. 2a of the manuscript), we can reconstruct the plasma fields based on the model given by [1]. In fact, this model can be simplified as follows, if neglecting the magnetic fields,

$$I_{wake}(y,z) = \frac{\delta n}{n_0} \simeq \kappa\epsilon_0 \nabla \cdot \int_{-\infty}^{+\infty} \mathbf{E}(z-x,x,y)dx = \kappa \int_{-\infty}^{+\infty} \rho_p(z-x,x,y)dx \qquad (1)$$

where I_{wake} represents the probe density modulation after crossing the full transverse extent of the wakefield and ρ_p represents the plasma charge density with $\rho = 0$ meaning that the plasma is neutralized. $\kappa = eL/Mcp_0\epsilon_0$ and all other parameters are defined the same as in the Methods and Materials of the manuscript. Eq. S1 shows that I_{wake} is simply an integral of the plasma density along 45 degree. Since the drive bunch behaves like an

* yang.wan@weizmann.ac.il

ultra-thin charged disk moving at light speed, the plasma density of the linear wake has a simple form close to $\sin(k_p(z-ct))G(x)H(y)$ [2, 3], where k_p is the plasma wave number and $G(x)H(y)$ resembles the bi-Gaussian lateral profile of the driver. As a result, Eq. S1 is further transformed into,

$$I_{wake}(y,z) \simeq -\sqrt{2\pi}\kappa \mathbf{e}^{-(k_p\Delta_x)^2/2}\Delta\rho_0\Delta_x \sin(k_p z)H(y) \tag{2}$$

Here we define the start point of the wakefield (the driver position) as $z=0$ and no wakefield at $z>0$. Δ_x is the horizontal RMS size of the driver and $\Delta\rho_0$ is the density perturbation amplitude of the wakefield. k_p, Δ_x and $\Delta\rho_0$ can thus be simply estimated based on Eq. S2 with the probed wakefield images.

We now consider the probe electrons that traverse the driver. Since they do not pass through the full extent of the wakefield, the corresponding density modulation is slightly different from Eq. S1, $\widetilde{I_{wake}}(y,z) \simeq \kappa\int_z^{+\infty}\rho(z-x,x,y)dx$. This integral can be numerically solved and the obtained $\widetilde{I_{wake}}(y,z)$ for $z>0$ (preceding the plasma wake) has a similar form as the bi-Gaussian function $G(z)H(y)$ (Though the distribution along z is slightly different). Given that the contribution of wakefield to the probe deflection is still much less than that of the beam self-fields, we simplify the above relation as,

$$\widetilde{I_{wake}}(y,z) \simeq \kappa\sigma_{w0}G(z)H(y) \tag{3}$$

where $\sigma_{w0} = \int_0^{+\infty}\Delta\rho_0\sin(k_p x)G(x)dx \simeq \Delta\rho_0(k_p\Delta_x^2)\mathbf{e}^{-(k_p\Delta_x)^2/2}$.

II. THE EFFECT OF PLASMA SCREENING ON THE PROBE DENSITY MODULATION

When an ultra-relativistic electron bunch propagates in a plasma (pre-ionized by the residual laser in our case), it pushes outwards the plasma electrons that form the trailing wakefield. For a typical LPA electron bunch, its pulse duration (i.e., a few femtoseconds) is usually shorter than the plasma wavelength (10s μm for density below 10^{18} cm^{-3}). The plasma electrons thus experience an instantaneous impulse in this transverse direction. For the driver's charge of tens of pC, this impulse can be very intense such that the electrons are accelerated up to relativistic speeds. As a consequence, lateral plasma currents increase rapidly within the drive bunch, which produce transverse electromagnetic fields against the

space-field fields from the bunch. This plasma screening phenomenon is observed in our PIC simulation when comparing the transverse E fields before and after the bunch enters the plasma (see Fig. S4 and section III), and was partially discussed in Ref [2, 4], whereas no clear analytical model was presented to estimate the relevant fields. It is noted that the bunch because of divergence also contains radial currents that could introduce similar effects as the plasma currents. However the generated fields over the main beam fields are on the same order of the bunch divergence ($< 10^{-2}$) and thus this effect is neglected here for simplicity.

In the following, we derive a simple model for the plasma screening to an ultra-short bunch based on the Maxwell equations and linear wakefield theory. In the Lorenz gauge, the potential equations are,

$$\left(\nabla^2 - \frac{1}{c^2}\frac{\partial^2}{\partial t^2}\right)\phi = -\frac{\rho_p + \rho_b}{\epsilon_0}$$
$$\left(\nabla^2 - \frac{1}{c^2}\frac{\partial^2}{\partial t^2}\right)\mathbf{A} = -\mu_0(\mathbf{J}_p + \mathbf{J}_b)$$
$$\nabla \cdot \mathbf{A} = -\frac{1}{c^2}\frac{\partial \phi}{\partial t}$$

where μ_0 is the vacuum permeability, $\mathbf{A}$ and ϕ represent the vector and scalar potentials, respectively, ρ_p and ρ_b represent the plasma and beam charge densities, respectively, $\mathbf{J}_p$ and $\mathbf{J}_b$ represent the plasma and beam current densities respectively. In the quasi-static approximation, the coordinates $(x,\, y,\, z,\, t)$ are substituted with $(x,\, y,\, \xi = ct - z)$ based on the assumption of non-evolving driver. The above equations then become,

$$\nabla_\perp^2 \phi = -\frac{\rho_p + \rho_b}{\epsilon_0} \tag{4}$$
$$\nabla_\perp^2 \mathbf{A} = - - \mu_0(\mathbf{J}_p + \mathbf{J}_b) \tag{5}$$
$$\nabla_\perp \cdot (cA_\perp) = -\frac{\partial \psi}{\partial \xi} = E_z \tag{6}$$

where $\psi = \phi - cA_z$. Since the drive bunch is very short, it is reasonable to assume over the small bunch length, that the plasma electrons only gain a lateral momentum with no longitudinal motion, and that the plasma density is not perturbed, i.e., $J_{p,z} \approx 0$ and $\rho_p \approx 0$. Neglecting the bunch's transverse divergence, it is then straightforward to conclude that, in this region, ϕ results from the bunch's charge density ρ_b, A_z from the bunch's longitudinal current density $J_{b,z}$, and $A_\perp$ from the plasma lateral current density $J_{p,\perp}$. Transverse $E_\perp$

and $B_\perp$ are then calculated from the potentials as, $E_\perp = -\nabla_\perp \phi - c\partial A_\perp/\partial\xi$ and $B_\perp = \nabla_\perp \times A_z + \partial A_\perp/\partial\xi$, where the first terms on the right hand of both formulas defined as $E_{b,\perp}$ and $B_{b,\perp}$ come from the drive bunch and the second terms defined as $E_{p,\perp}$ and $B_{p,\perp}$ come from the plasma lateral currents.

From the PIC simulation, it is shown that the shape of $E_{p,\perp}$ is close to the bunch self-fields both longitudinally and transversely. Assuming the drive bunch 3D distribution as a tri-Gaussian shape and Differentiating Eq. S6 by ξ, one can obtain the peak value of $E_{p,\perp}$ as $E_{p,\perp 0} = \sqrt{2/\pi}(k_p\delta_z)E_{z0}$, where δ_z is the bunch longitudinal RMS size and E_{z0} is the maximum plasma longitudinal field. Since E_{z0} can be easily calculated from $\Delta\rho_0$ in linear wakefield theory. As a result, the ratio between $E_{p,\perp}$ and $E_{b,\perp}$ is expressed as,

$$\frac{E_{p,\perp}}{E_{b,\perp}} \simeq 2\pi k_p \chi \frac{\Delta\rho_0}{\sigma_{b0}} \tag{7}$$

where Δ_x and Δ_y represent the bunch's transverse RMS sizes, σ_{b0} is the bunch's peak surface charge density and $\chi = \frac{\Delta_x^2\Delta_y^2}{\Delta_x^2 + \Delta_y^2 + k_p^2\Delta_x^2\Delta_y^2}$. Using the same derivation as Eq. 1 of the mansucript, the probe density modulation caused by the plasma screening is then,

$$I_{screen}(y,z) \simeq \kappa\sigma_{s0}G(z)H(y) \tag{8}$$

where $G(z)$ and $H(y)$ are defined the same as the previous section, and $\sigma_{s0} = 2\pi k_p\chi\Delta\rho_0$ is obtained directly from Eq. S7.

Taking these two effects into consideration, the total probe density modulation can thus be simply expressed as,

$$I_{total}(y,z) \simeq \kappa(\sigma_{b0} + \sigma_{w0} + \sigma_{s0})G(z)H(y) \tag{9}$$

where σ_{w0} and σ_{s0} can be obtained from Eq. S3 and Eq. S8 respectively, and σ_{b0} represents the bunch peak surface charge density and can be obtained from Eq. 1 of the manuscript. Since it is apparent that σ_{b0} is negative and both σ_{w0} and σ_{s0} are positive, the bunch charge would be underestimated without considering these two effects. Eq. 2 of the manuscript is a general form of Eq. S9.

III. 3D PIC SIMULATION COMPARISON WITH THE EXPERIMENT

From the experimental probe image of Fig. 2a of the manuscript, we can calculate the electron bunch's lateral distribution. Fitting the elliptical structure with a bi-Gaussian

profile, the RMS sizes of the vertical and horizontal are estimated as $\Delta_y = 7.5\ \mu$m and $\Delta_x = 12.5\ \mu$m respectively. For estimating the beam charge, we need to take into account the above two effects. First, from the plasma wakefield image captured by the probe, the plasma density perturbation can be obtained based on Eq. S2. Afterwards, it is straightforward to get the value of σ_{w0} as 18 ± 2 fC μm^{-2} based on Eq. S3. Meanwhile, σ_{s0} can be calculated as 30 ± 3 fC μm^{-2} from the plasma screening formula Eq. S8. As a result, the peak surface charge density of the bunch σ_{b0} is obtained as -160 ± 17 fC μm^{-2} from Eq. S9. The overall charge of the investigated electron bunch is thus calculated as 94 ± 10 pC with the error bar arising from the probe peak energy fluctuation and the uncertainties of probe/probed electron beams duration (see Methods and Materials).

To directly compare with the above experimental data, we have performed a 3D PIC simulation using FBPIC [5] with solely an electron bunch propagating in a plasma of the density 2.5×10^{16} cm^{-3}. The reason why the laser was not included is that a separate PIC simulation showed that the laser intensity at this position was already quite weak such that the generated wakefield strength was only one eighth of the beam driven wakefield. The bunch was set to have a tri-Gaussian distribution with the RMS sizes as $\Delta_x = 12.5$ μm (horizontal), $\Delta_y = 7.5\ \mu$m (vertical) and $\delta_z = 1\ \mu$m (longitudinal) respectively. The energy spectrum contained a peak energy of 150 MeV with 45% FWHM spread (the same as experiment) and the overall charge was 94 pC. For simplicity, the bunch was set with no transverse divergence. More details about the simulation setup can be found in the Methods and Materials.

After propagating for around 300 μm in plasma, the electron bunch launches a stable quasi-linear plasma wave, where the on-axis electron density is around 7×10^{15} cm^{-3} (see Fig. S3). In Fig S4, we also compare the E_y field before and after the electron bunch enters the plasma, and one can see the E_x within the bunch is reduced by 12% (see Fig. S4c).

At this moment, an electron probe (the same parameters as Fig.2 of the manuscript) traverses the electron bunch and the associated wakefield along the x axis. After drifting 13 mm, the generated density modulation is plotted in Fig. S5a. Fitting the elliptical hollow structure with a bi-Gaussian function, we obtain the bunch's transverse RMS sizes as $\Delta_x = 13.9\ \mu$m and $\Delta_y = 8.4\ \mu$m respectively, which are slightly larger than the actual beam sizes by 12% because some of the probe electrons start to cross other's traces. Meanwhile, the estimated peak surface charge density of the bunch is slightly lower than the experi-

mental one. As a result, the overall charge based on the analytical model gives 98 pC, in good agreement with the input value of 94 pC. Without considering the plasma effects, the estimated charge is lower by 29%.

It is noted that with even longer drift distance (stronger density modulation) the estimated total charge increases very slightly, whereas the sizes of elliptical structures become larger due to the trajectory expansion of the probe particles. Therefore, in order to get a reasonable size measurement (deviation less than 20%), the probe relative density modulation is better to satisfy $|I_{total}| < 0.5$.

---

[1] CJ Zhang, JF Hua, XL Xu, F Li, C-H Pai, Y Wan, YP Wu, YQ Gu, WB Mori, C Joshi, et al. Capturing relativistic wakefield structures in plasmas using ultrashort high-energy electrons as a probe. *Scientific reports*, 6(1):1–9, 2016.

[2] W Lu, C Huang, MM Zhou, WB Mori, and T Katsouleas. Limits of linear plasma wakefield theory for electron or positron beams. *Physics of plasmas*, 12(6):063101, 2005.

[3] Eric Esarey, CB Schroeder, and WP Leemans. Physics of laser-driven plasma-based electron accelerators. *Reviews of modern physics*, 81(3):1229, 2009.

[4] R Lehe, Cédric Thaury, Agustin Lifschitz, J-M Rax, and Victor Malka. Transverse dynamics of an intense electron bunch traveling through a pre-ionized plasma. *Physics of Plasmas*, 21(4):043104, 2014.

[5] Rémi Lehe, Manuel Kirchen, Igor A Andriyash, Brendan B Godfrey, and Jean-Luc Vay. A spectral, quasi-cylindrical and dispersion-free particle-in-cell algorithm. *Computer Physics Communications*, 203:66–82, 2016.

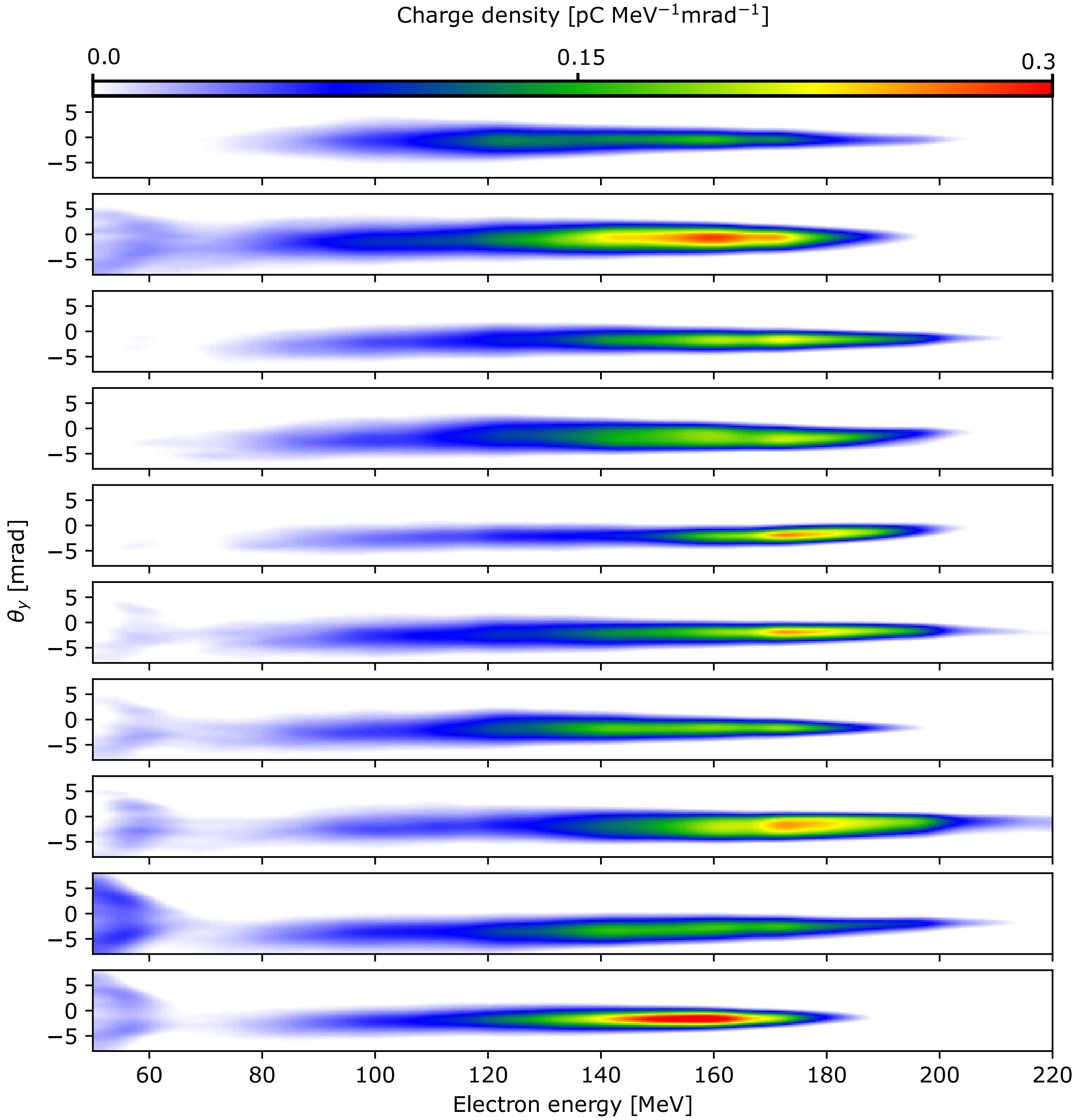

FIG. S1. Energy spectra of the investigated electron beams generated from the laser wakefield in ten consecutive shots.

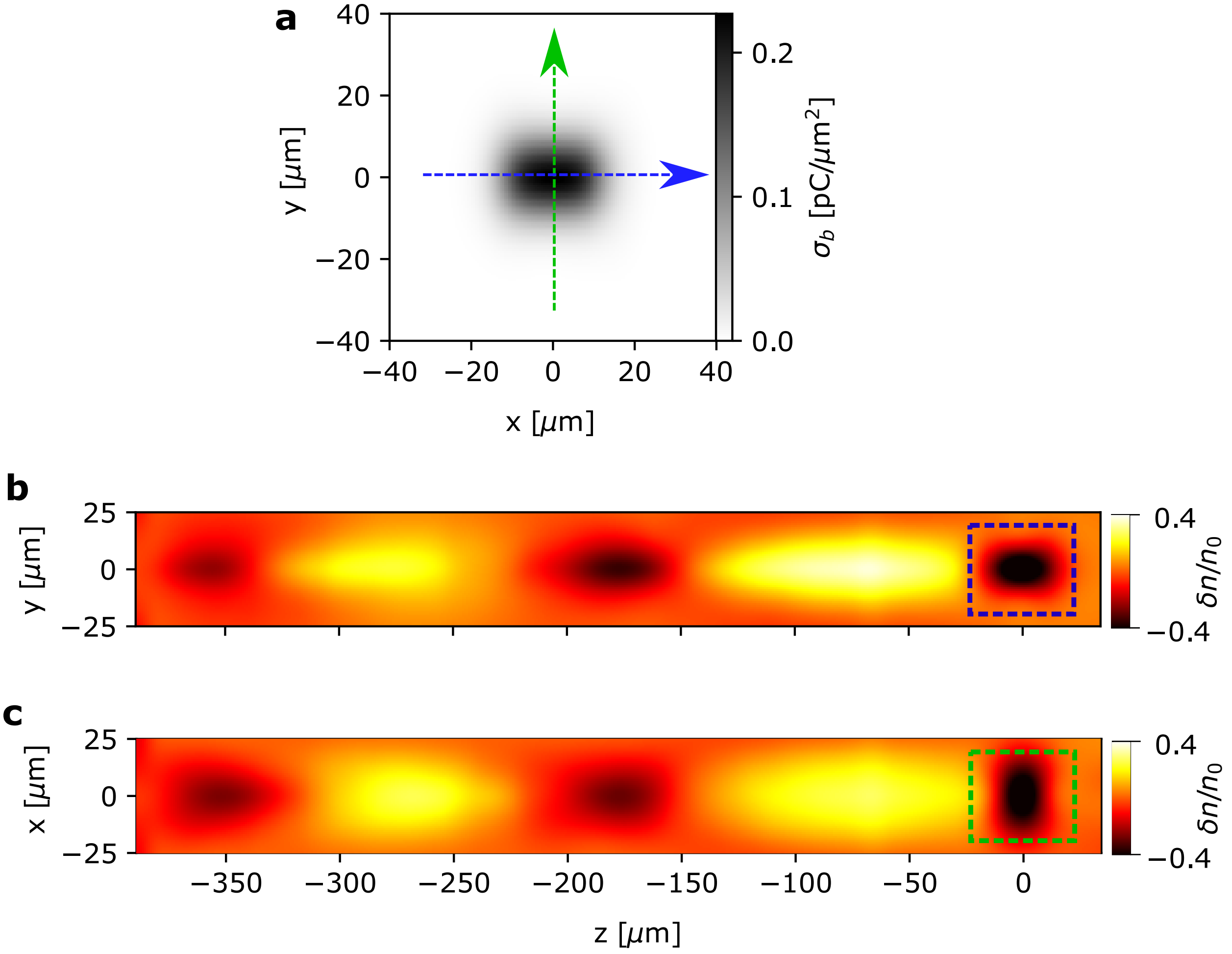

FIG. S2. (a) The lateral profile of the investigated electron beam accelerated in the simulation shown in Fig.2 of the manuscript. Two arrows (blue and green) represent the directions of the probe as it traverses the electron beam respectively. (b) (c) Simulated probe images for horizontal (b) and vertical (c) orientations. The plasma wakes propagates from left to right and ahead of them is the driven electron beam (marked by the dashed box).

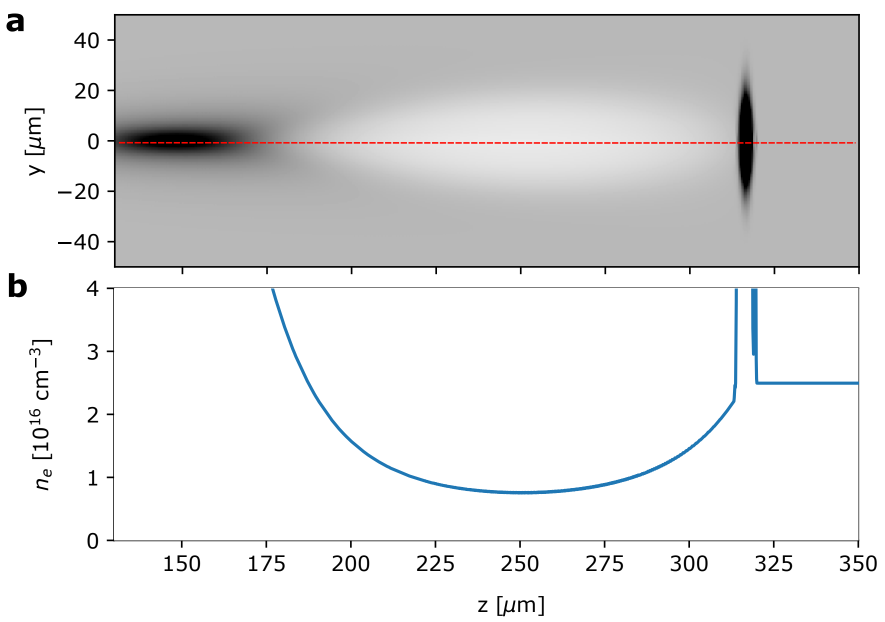

FIG. S3. (a) The simulated electron density n_e distribution of a plasma wake driven by a short electron bunch.(b) The on-axis lineout of (a). The beam and plasma parameters are summarized in Section III of the Supplementary.

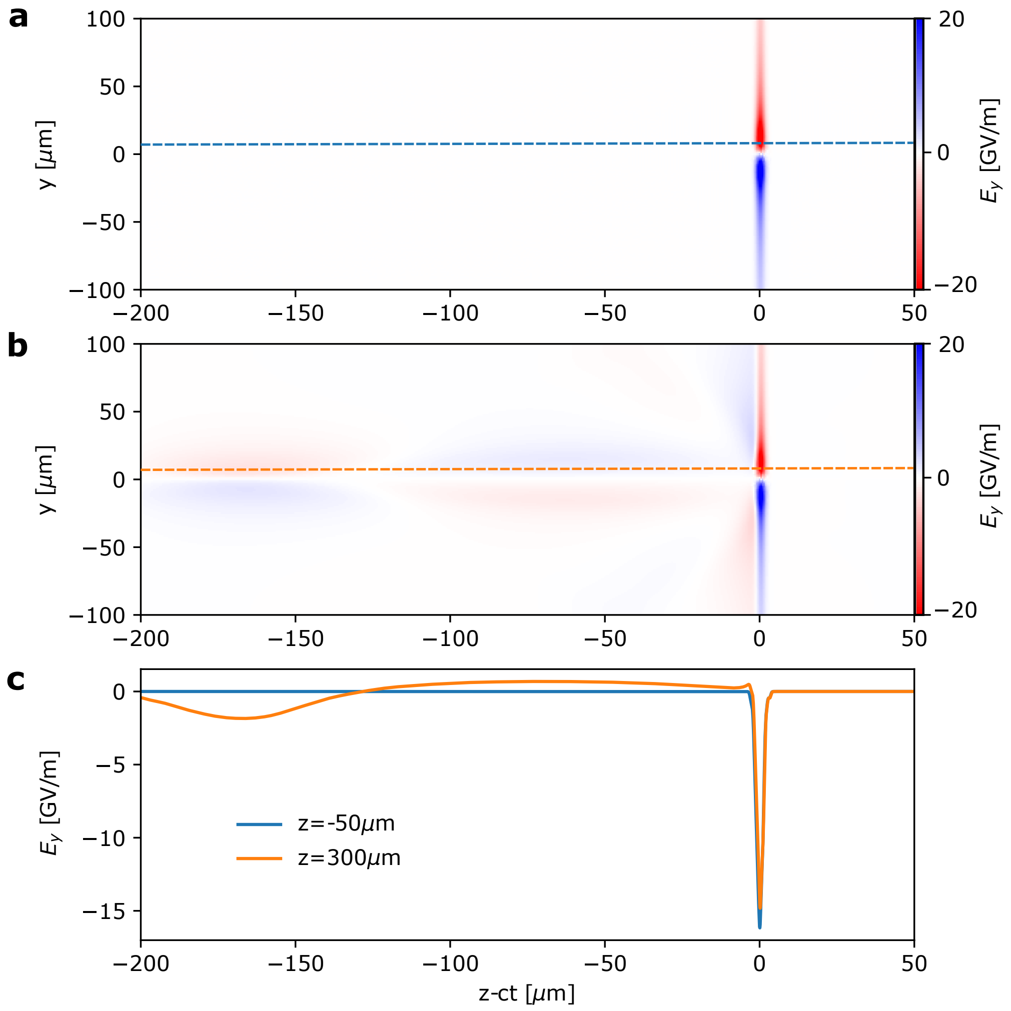

FIG. S4. (a) The simulated transverse electric field E_y at $z = -50$ μm where the electron bunch is still in vacuum. (b) The simulated transverse electric field E_y at $z = 300$ μm where the electron bunch is in plasma. (c) The longitudinal lineouts of E_y along the dashed lines of (a) and (b), respectively. The beam and plasma parameters are summarized in Section III of the Supplementary.

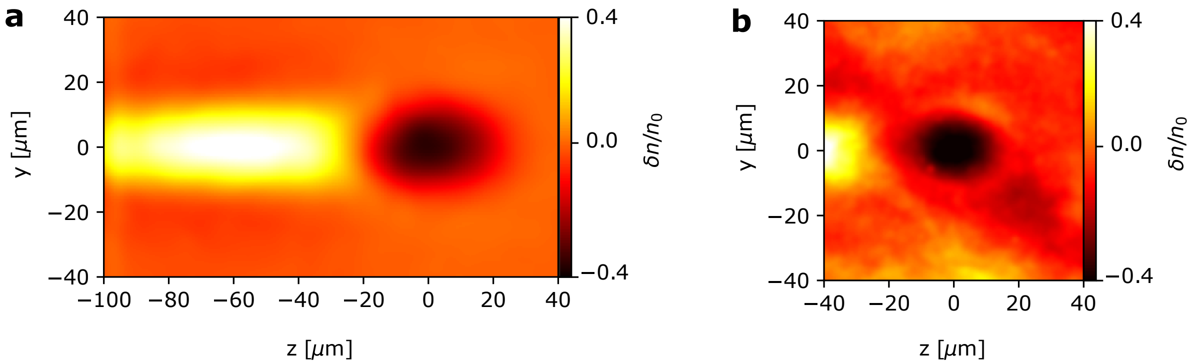

FIG. S5. (a) The simulated probe image of an electron beam propagating in a low-density plasma. (b) The experimental probe image extracted from Fig. 2f of the manuscript. The simulation parameters are summarized in Section III of the Supplementary.
